# Structure Learning in a Sensorimotor Association Task

**DOI:** 10.1371/journal.pone.0008973

**Published:** 2010-01-29

**Authors:** Daniel A. Braun, Stephan Waldert, Ad Aertsen, Daniel M. Wolpert, Carsten Mehring

**Affiliations:** 1 Bernstein Center for Computational Neuroscience, Freiburg, Germany; 2 Faculty of Biology, Albert-Ludwigs-Universität, Freiburg, Germany; 3 Department of Engineering, University of Cambridge, Cambridge, United Kingdom; The University of Western Ontario, Canada

## Abstract

Learning is often understood as an organism's gradual acquisition of the association between a given sensory stimulus and the correct motor response. Mathematically, this corresponds to regressing a mapping between the set of observations and the set of actions. Recently, however, it has been shown both in cognitive and motor neuroscience that humans are not only able to learn particular stimulus-response mappings, but are also able to extract abstract structural invariants that facilitate generalization to novel tasks. Here we show how such structure learning can enhance facilitation in a sensorimotor association task performed by human subjects. Using regression and reinforcement learning models we show that the observed facilitation cannot be explained by these basic models of learning stimulus-response associations. We show, however, that the observed data can be explained by a hierarchical Bayesian model that performs structure learning. In line with previous results from cognitive tasks, this suggests that hierarchical Bayesian inference might provide a common framework to explain both the learning of specific stimulus-response associations and the learning of abstract structures that are shared by different task environments.

## Introduction

Since the heyday of behaviourism, stimulus-response theories of learning are a central theme in the theoretical neuroscience of learning and have successfully explained a wide range of experimental data in animal and human learning [1]. In particular, classical conditioning as propounded by Pavlov and Skinner's operant conditioning pioneered the concept that an animal's adaptive behaviour is based on associations between sensory stimuli and motor responses [2]–[4]. Pavlov believed that ultimately all of animal and human behaviour would be explained on the basis of stimulus-response associations. Later, Rescorla and Wagner formalized such associative learning in a very simple and powerful learning rule [5], [6] that explains a vast array of experimental effects. In fact, the Rescorla-Wagner rule can be considered as a form of a previously suggested learning rule, the delta-rule, that can be used to train simple neural networks [7]. More recent neural network models such as back-propagation [8], [9] and basis function networks [10], [11] are simply non-linear extensions of the originally proposed models in the sense that they implement a mapping from stimulus to motor response by adapting (synaptic) weights in networks with fixed topology. Similarly, most reinforcement learning schemes [12] seek to learn environment-specific stimulus-response contingencies, rather than more abstract adaptive policies that can cope with a variety of different environments.

Critics were quick to point out that stimulus-response theories of learning liken the nervous system to some kind of “complicated telephone switchboard” [13] that continuously transforms impinging sensory stimuli into motor responses. Learning in such a switchboard consists of strengthening and weakening the connections between input relays and output units. Cognitive scientists and psychologists have pointed out that many animal behaviours seem to transcend simple associative learning [14], [15], for example the learning of mental maps [13], insightful learning [16] and abstract rule learning [17]. Unfortunately, though, these alleged types of ‘higher order’ learning have often resisted mathematical formalization. Recent progress in the field of Bayesian learning, however, suggests that some ‘higher order’ learning phenomena in cognitive science and neuroscience could be explained by the process of structure learning.

In contrast to parametric learning that is usually studied, structure learning is not concerned with learning the particular contingencies of a single task, for example, a particular stimulus-response relationship. Rather, structure learning can be regarded as a process of abstraction that extracts general invariants [18]. In this way, general forms of a rule can be learned that are widely applicable to a possibly large set of related tasks. Such structure learning has been recently reported both in cognitive [19]–[25] and motor neuroscience [26], [27], [18]. Here we study structure learning in a sensorimotor association task.

## Results

**Figure 1:**
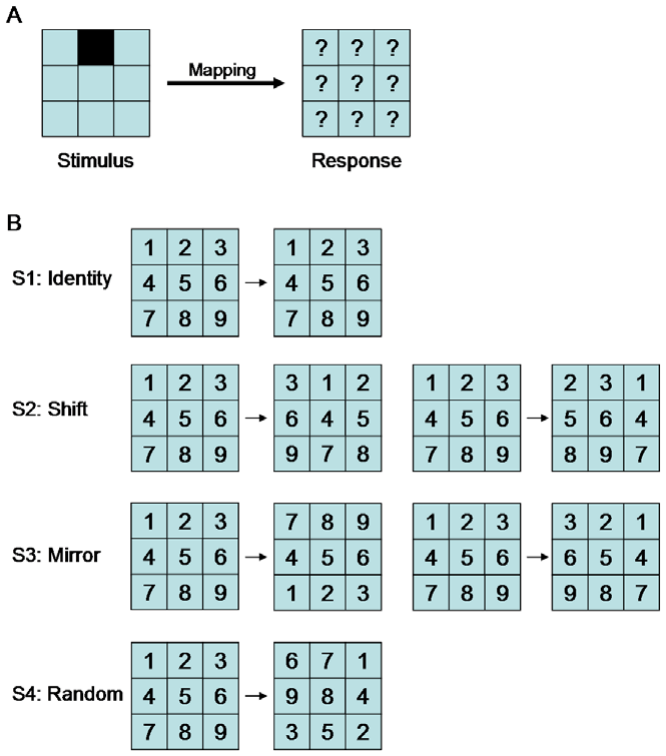
Task description. (A) Subjects had to learn a mapping from a 3×3 stimulus board to a 3×3 action board. The stimulus was presented by lighting up one of the nine squares. The subject then had to press one of the nine response buttons associated to that stimulus. (B) There were six possible mappings with four different structures (S1 to S4). The identity and the random structure comprised only one mapping each. The shift structure consisted of a right-shift and a left-shift mapping. The mirror structure consisted of a horizontal and vertical mirror mapping.

To investigate features of structure learning, we exposed subjects to a stimulus-response learning task, where the stimulus-response patterns were characterised by different structural constraints. Subjects were presented with nine possible stimuli and could respond with one of nine possible actions (see Figure 1A and Methods for details). This defines a set of nine pairs of stimuli and their associated correct responses, resulting in 362,880 (9!) possible one-to-one sensorimotor mappings. Subjects had to learn six different mappings that were characterised by four different structural features: (1) an identity mapping that constitutes the baseline mapping, as it is most readily learned, (2) two shift mappings, where the correct response was shifted either to the right or to the left compared to the identity mapping, (3) two mirror mappings, where the correct response was mirrored around the vertical or horizontal axis, again compared to the identity mapping, and (4) a random mapping where stimuli and responses were not associated by any apparent rule (see Figure 1B). We counted the number of trials it took subjects to learn any of the mappings to assess their performance.

**Figure 2:**
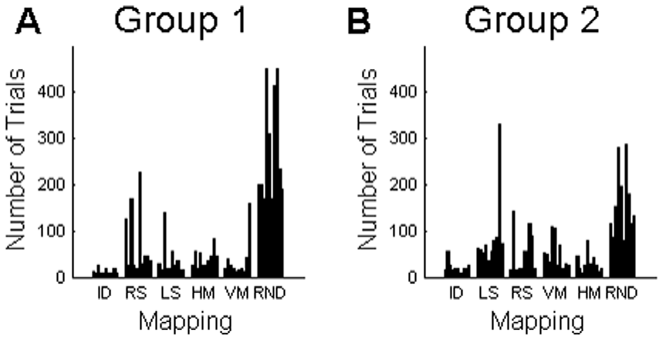
Numbers of trials required by subjects to learn the different mappings. (A) The first group learned the right shift before the left shift and the horizontal mirror before the vertical mirror. (B) The second group learned the two versions of the shift and mirror mappings in reverse order. Each group had 10 subjects. Statistical comparisons between the different mappings in each group can be found in Tables 1 and 2, and comparisons between the groups in Table 3. ID = Identity mapping. RS = Right shift mapping. LS = Left shift mapping. HM = Horizontal mirror mapping. VM = Vertical mirror mapping. RND = Random mapping.

Importantly, there were two groups of subjects that learned the two shift mappings and the two mirror mappings in reversed order, i.e. the first group went from right-shift to left-shift and from horizontal to vertical mirror and the second group went from left-shift to right-shift and from vertical to horizontal mirror. Since both of the shift mappings shared the shift structure and both of the mirror mappings shared the mirror structure, we hypothesized that learning one mapping (e.g. right-shift mapping of the shift structure) would subsequently facilitate learning of the other mapping with the same structure (e.g. left-shift). To assess this hypothesis we analysed the number of learning trials in the two groups – data shown in Figure 2.

**Figure 3:**
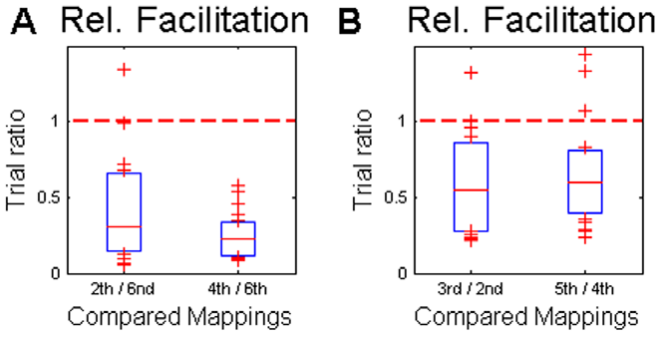
Relative facilitation of learning. (A) Mappings with structural constraints were learned much faster than the random mapping. (B) Learning the third (fifth) mapping was facilitated in both groups compared to learning the second (fourth) mapping. Shown are the medians and the lower and upper quartiles of the trial ratios of all subjects and the average has been taken over both groups.

As expected, learning the identity mapping was in most cases faster than learning any of the other mappings – compare Table 1 and Table 2. Similarly, learning the random mapping was in most cases much slower than learning any of the structured mappings (see Table 1 and Table 2), which suggests that mappings with structural constraints are learned more readily than mappings without any obvious structure. We also computed for each subject the ratio between the trials required for learning the random mapping and the trials required for learning the first shift and the first mirror mapping (Figure 3A). The median of all the ratios was significantly smaller than unity (p<0.01, Wilcoxon signed rank test), which again implies faster learning of the structured mappings.

**Table 1:** P-values of paired Wilcoxon signed rank test for comparing the number of learning trials of two different mappings in Group 1.

Group 1	ID	RS	LS	HM	VM	RND
**ID**	-	*0.002	*0.004	*0.006	0.016	*0.002
**RS**	-	-	*0.004	0.91	0.16	*0.006
**LS**	-	-	-	0.287	0.797	*0.002
**HM**	-	-	-	-	0.131	*0.002
**VM**	-	-	-	-	-	*0.002
**RND**	-	-	-	-	-	-

The highlighted fields show significant differences (P<0.01) between learning two mappings. ID = Identity mapping. RS = Right shift mapping. LS = Left shift mapping. HM = Horizontal mirror mapping. VM = Vertical mirror mapping. RND = Random mapping.

**Table 2:** P-values of paired Wilcoxon signed rank test for comparing the number of learning trials of two different mappings in Group 2.

Group 2	ID	RS	LS	HM	VM	RND
**ID**	-	*0.002	0.098	0.016	0.397	*0.002
**RS**	-	-	0.16	0.232	0.027	0.084
**LS**	-	-	-	0.898	0.275	*0.01
**HM**	-	-	-	-	0.137	*0.002
**VM**	-	-	-	-	-	*0.002
**RND**	-	-	-	-	-	-

The highlighted fields show significant differences (P<0.01) between learning two mappings. ID = Identity mapping. RS = Right shift mapping. LS = Left shift mapping. HM = Horizontal mirror mapping. VM = Vertical mirror mapping. RND = Random mapping.

**Table 3:** P-values of paired Wilcoxon ranksum test for comparing the number of learning trials of different mappings between Group 1 (G1) and Group 2 (G2).

	G1 vs. G2
**ID**	0.021
**RS**	0.13
**LS**	0.382
**HM**	0.649
**VM**	0.649
**RND**	0.014

ID = Identity mapping. RS = Right shift mapping. LS = Left shift mapping. HM = Horizontal mirror mapping. VM = Vertical mirror mapping. RND = Random mapping.

Interestingly, we also found within-structure facilitation effects. For example, learning the second instance of the shift mapping (e.g. left shift mapping in the first group) proceeded much faster in most subjects than learning the first instance (e.g. right shift mapping in the first group). Accordingly, the ratio of learning trials between the second and the first occurrence of the shift mapping was significantly below unity (p<0.01, Wilcoxon signed rank test), which implies facilitation of learning for the second mapping (Figure 3B). Since the two groups experienced the two shift mappings in reversed order, this facilitation cannot be accounted for by an intrinsic simplicity of either one of the two shift mappings. This suggests that by experiencing the first instance of a shift mapping, subjects have learned something general about shift mappings that facilitated learning of the second instance. Furthermore, we observed a similar facilitation pattern for learning mirrored mappings, as the ratio between the second and the first occurrence of the mirror mapping was also significantly below unity (p<0.02, Wilcoxon signed rank test).

**Figure 4:**
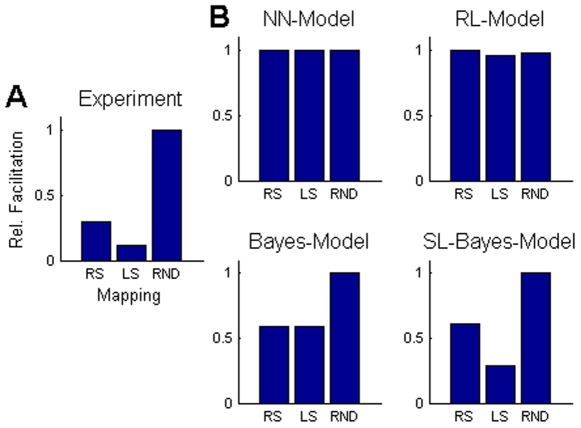
Modelling of the facilitation effect. (A) The experimental data shows a strong facilitation of learning a structured mapping (right-shift or left-shift) compared to a random mapping (RND). In addition, there is also a strong facilitation from learning the first instance of a shift mapping to learning the second instance. (B) The feed-forward neural network (NN) and the reinforcement learning (RL) model show no facilitation effects. The non-hierarchical Bayesian model shows a facilitation effect for the structured mappings if the prior probabilities of these mappings are elevated. The structure learning (SL) Bayes model shows both facilitation effects, because by learning the first mapping the posterior over structures assigns more probability to all other mappings with the same structure. All plots show median values, for the model these were computed over 100 simulation runs.

To test whether these results could be explained by merely learning stimulus-response associations we employed four different learning models to reproduce the observed facilitation effects. First, we used a simple feed-forward neural network to regress the different mappings. This translates our task into a supervised learning problem. To examine the relative speed of learning we used the number of trials taken to learn the random mapping as a normalising factor (i.e. number of trials for a random mapping was taken as unity). We initialized the network with the identity mapping before learning either the right-shift mapping or the random mapping. No facilitation was observed for learning the shift mapping (Fig. 4B, NN-model). We then initialized the network for the right-shift mapping before learning the left shift mapping in order to study whether learning a right-shift might facilitate learning a left-shift. Again there was no facilitation (Fig. 4B, NN-model). We also used a simple reinforcement learning model that learned the mappings from only binary feedback, i.e. reward 1 if the correct action was chosen, and reward 0 otherwise (see Methods for details). Actions were chosen according to their value from a softmax-rule, and the action values were updated using the Rescorla-Wagner rule. We performed the same three experiments as in the neural network case and again found no facilitation (Fig. 4B, RL-model).

**Figure 5:**
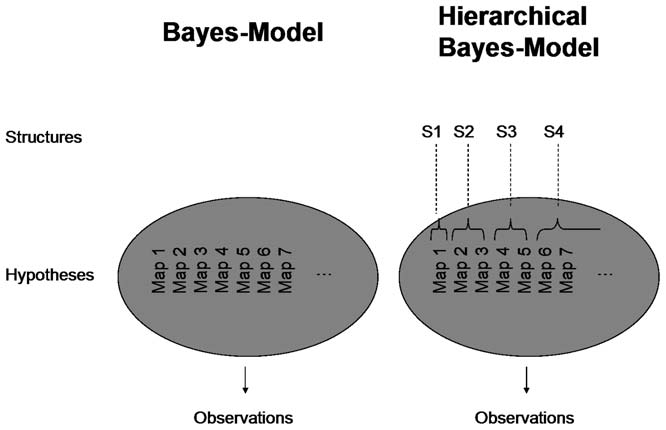
Graphical Model of the non-hierarchical and the hierarchical Bayesian model. In the non-hierarchical model the observations provide evidence for each hypothesis separately. In the hierarchical model the observations not only provide evidence for the hypotheses, but also for the different structures (which in turn might shift some evidence to structure-compatible hypotheses).

In Bayesian models, the speed of learning a particular hypothesis can be influenced by the setting of the prior. We therefore devised two Bayesian models to account for the observed facilitation effects – compare Figure 5. We used a standard, non-hierarchical Bayesian model where the set of hypotheses was given by the set of all possible mappings. We assigned a higher prior probability to all the structured mappings considered in this study (i.e. shifts and mirrors). Accordingly, such a model can account for facilitated learning of structured mappings compared to the random mapping (Fig. 4B, Bayes model). However, this model fails to capture the effect of facilitated learning of the second instance of a structure compared to learning the first instance (e.g. facilitated learning of the left-shift when preceded by a right-shift). Therefore, we constructed a hierarchical Bayesian model that not only does inference over different hypotheses, but also maintains a probability distribution over different structures. Thus, after learning a particular hypothesis that is part of a certain structure (e.g. the right-shift hypothesis of the shift structure) the probability of that structure is increased. Then, after learning the right-shift structure the learning of all shift structures is facilitated, because the prior reflects an increased probability of encountering shift structures. The hierarchical Bayesian model is therefore able to account for both facilitation effects (Fig. 4B, SL-Bayes model).

**Figure 6:**
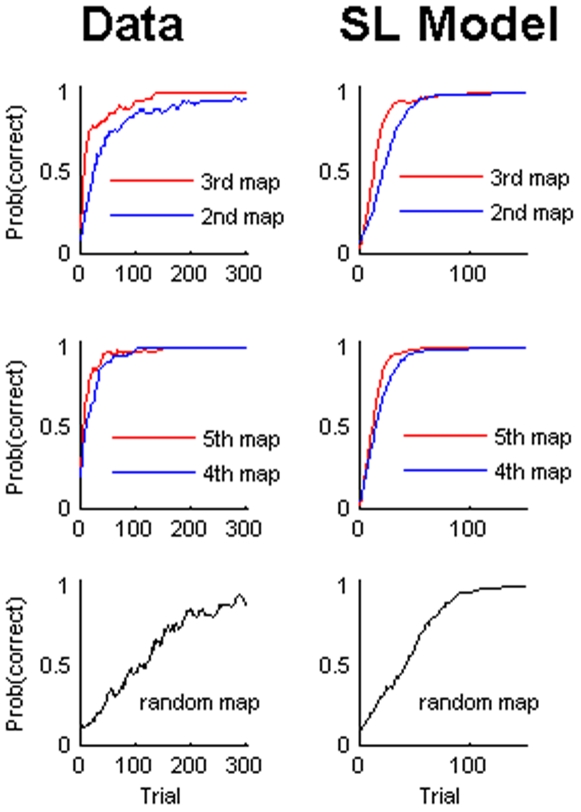
Trial-by-trial evolution of learning. For the experimental data we averaged over subjects to compute the probability that the correct action was chosen on the basis of the fraction of subjects that chose the correct action in each trial. For the model we determined the probability of choosing the correct action by computing the probability of choosing the correct action given the action and observation stream of each subject and again averaged over subjects. All curves were smoothed with a Savitzky-Golay-Filter of polynomial order 1 and length 11.

Moreover, we investigated model predictions of how learning proceeds over trials. We fed the Bayesian model with the action and observation stream from subjects and computed the probability the model would assign to choosing the correct action given the subject's evidence – compare Fig. 6. When initializing the Bayesian model with the appropriate priors (as above, see Methods), both facilitation effects become visible in the response curves over trials. Learning a shift mapping facilitates learning a second shift mapping (2^nd^ and 3^rd^ map in the upper panels of Fig. 6), learning a mirror mapping facilitates learning a second mirror mapping (4^th^ and 5^th^ map in the middle panels of Fig. 6), and learning a random mapping is always slower than learning any of the structured mappings (lower panels in Fig. 6). These facilitation effects are also visible in the empirical frequencies of choosing the correct action as exhibited by subjects (compare Fig. 6. left panels). To compute these empirical probabilities of action selection we determined the fraction of subjects that chose the correct action in any one trial. While there is a good qualitative correspondence between data and model for the dynamics of learning, it is important to note that the number of trials required to achieve comparable performance is very different. Especially, subjects take roughly double the number of trials for learning the random mapping compared to an ideal learner (compare Fig. 6. lower panels).

**Figure 7:**
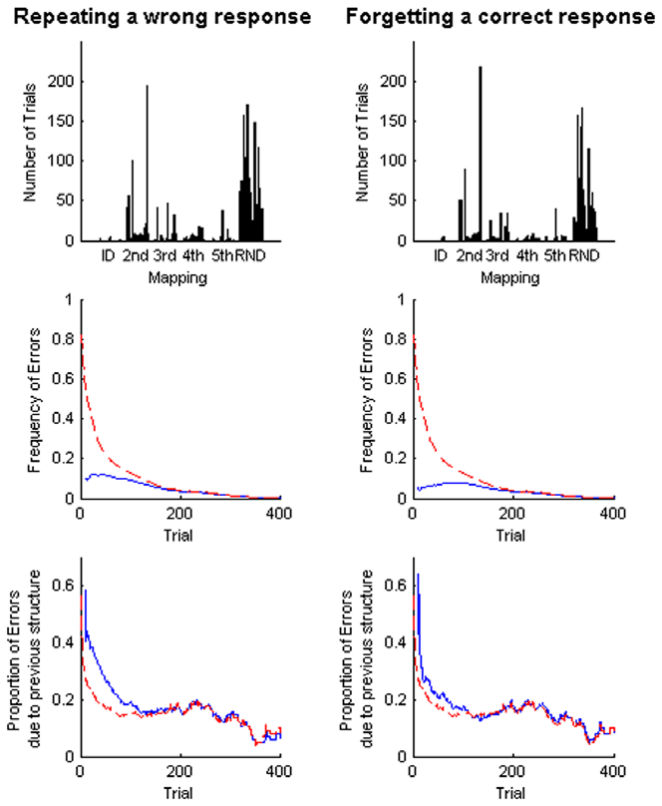
Forgetting errors in learning the different mappings. Subjects committed two kinds of errors that involved forgetting. The first kind of error (leftside panels) occurs when subjects repeat a wrong response to a stimulus that they had already seen. The second kind of error (rightside panels) occurs when subjects had already pressed the correct button once, but later on seem to have forgotten this correct response and pressed a different button when once more confronted with the same stimulus. The upper panels show the total number of errors committed by subjects when learning the different mappings. The middle panels show the probability of an error occurring in each trial following the first trial of a new mapping (averaged over all subjects and mappings, in red all false button presses, in blue the two specific kinds of error). The lower panels show the proportion of errors that can be explained by stimulus-response patterns consistent with the previously learned structure (averaged over all subjects and mappings, in red proportion of all false button presses that can be explained by previous structure, in blue the proportion of the two specific kinds of error that can be explained by previous structure). The frequency histograms were smoothed over 50 trial windows by moving average.

**Figure 8:**
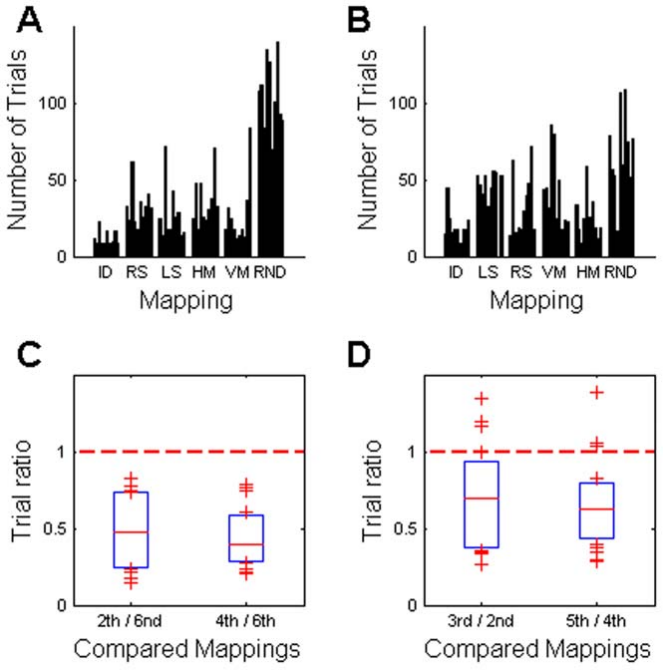
Facilitation effect in the absence of error trials. (A,B) Number of trials required by each subject to learn the mappings when disregarding all the error trials. (C,D) In the absence of error trials the facilitation effects remain all significant (p<0.02, Wilcoxon signed rank test).

To investigate possible sources of this difference, we examined whether subjects succumbed to errors due to forgetting which an ideal Bayesian actor would not suffer from. We considered two kinds of errors. We defined the occurrence of the first kind of error when a wrong response was repeated, i.e. when subjects gave the same wrong response to a stimulus that they had already seen. Clearly, an ideal actor would never repeat the same mistake. Furthermore, we defined the occurrence of the second kind of error when a correct response was forgotten, i.e. when subjects gave the wrong response to a stimulus that previously was answered correctly. Again, an ideal observer would not forget a correct response. We analysed the occurrence of these two kinds of errors when subjects learned the different mappings – see Fig. 7. Both kinds of errors occurred most frequently when learning a random mapping (p<0.01, Wilcoxon ranksum test), whereas there were practically no errors when learning the identity mapping. The numbers of both errors were also reduced when learning a shift mapping for the second time, if another shift mapping had been learned before (p<0.05, Wilcoxon ranksum test). For the mirror mappings the number of errors in the first and second exposure was not significantly different (p>0.05, Wilcoxon ranksum test). We also investigated the time course of errors and found that the probability of repeating a wrong response was elevated in early trials of learning a new mapping and that a high proportion of these repetition errors were consistent with the previously learned structure – see Fig. 7 (leftside panels). The time course of forgetting a correct response was qualitatively similar. However, the probability of forgetting the correct response was highest a bit later into learning a mapping – see Fig. 7 (rightside panels, blue lines). The occurrence of errors, however, did not explain the observed facilitation effects. Disregarding the error trials leaves the facilitation pattern qualitatively unchanged (Fig. 8), which ensures that the facilitation pattern is not exclusively due to forgetting. Thus, our Bayesian model, which does not include the process of forgetting, is apt to account for the observed facilitation effects presented in Fig. 1 and 8. However, the difference in time scales of learning observed in the experiment compared to the model predictions might be explained by the lack of forgetting in the model (compare Fig. 6).

## Discussion

In our experiments we found that human choice behaviour in a sensorimotor association task requires structure learning processes and cannot be accounted for by forming specific associations between sensory stimuli and motor responses. Many traditional learning schemes, like the Rescorla-Wagner rule or learning in feed-forward neural networks with fixed topology, have conceptualized sensorimotor learning as acquiring an association between a stimulus and the correct motor response. The facilitation effects we observed, however, suggest that humans learn much more than specific stimulus-response associations, namely that they also learn to extract abstract invariants that are applicable to a broad class of tasks. Learning a right-shift mapping, for example, facilitated learning a left-shift mapping in our task. Similarly, learning a left-shift mapping facilitated subsequent learning of a right-shift mapping. Therefore, our results cannot be explained by one of the two tasks being intrinsically easier than the other one. We observed a similar facilitation also for different versions of a mirror mapping. The only model that could explain this kind of facilitation was a hierarchical Bayesian model that takes probabilities over structures into account (e.g. shift structure), such that learning one instance of a structure can lead to higher prior probability of all the other instances of the same structure, thereby entailing facilitation. While the model provided a good qualitative fit to the observed facilitation effects, the time scales of the predictions were very different from those observed in the experiment. Subjects learned much slower than the Bayesian learner, at least partly due to the process of forgetting. Thus, in future it might be interesting to develop Bayesian models that include processes of forgetting.

Hierarchical Bayesian models have been previously proposed to account for structure learning effects in cognitive tasks, especially in causal reasoning [23], [22], [24], [28]. These previous studies focused on more complicated learning problems in which the higher-level inferences made through hierarchical Bayesian inference concern very abstract forms of knowledge, although there have also been studies that used Bayesian methods to explain causal inference in perception [29]. Here we show that the framework of Bayesian structure learning can explain facilitation effects in a simple sensorimotor association task. This is of particular interest, because Bayesian models have also been previously proposed to explain associative learning [30]–[32]. Thus, hierarchical Bayesian models might reconcile the idea of learning specific stimulus-response pairs with the idea of abstraction or structure learning. Learning specific stimulus-response pairs is instantiated by learning particular parameters for a specific mapping (a particular hypothesis), whereas structure learning also depends on updating probabilities over different structures that represent more abstract properties, such that learning a particular mapping also distributes probability mass to ‘structural neighbours’ that represent similar mappings.

In psychology, facilitation effects in visual discrimination experiments have been reported previously for learning intra-dimensional shifts compared to learning extra-dimensional shifts [33]–[36]. For example, when humans are trained using a stimulus set with a particular relevance dimension on which discriminations should be based on (e.g. shape), they adapt more rapidly to a novel stimulus set with the same relevance dimension (intra-dimensional shift), whereas they adapt more slowly when facing a novel stimulus set with a different relevance dimension (extradimensional shift, e.g. lines) [34]. Facilitation for intra-dimensional shifts has been interpreted as the ability to attend to the specific attributes of a stimulus and to use this information for learning novel discriminations. However, one could also interpret such facilitation as structural learning of abstract dimensions such as colour or shape.

In our experiments subjects could not discriminate explicit properties of the presented stimuli. Rather they had to extract abstract invariants or rules of the experienced stimulus-response mappings. In a Bayesian framework ‘discovering’ such rules means ‘finding’ the best-fitting structure and hypothesis in a given set of possible structures and hypotheses. This Bayesian account is entirely compatible with other rule-based approaches to concept learning [37], but a Bayesian estimator has to maintain a probability distribution over all alternatives at all times. Therefore, discovering a ‘new’ rule is only possible if this rule has been considered already as a possibility in the prior. Furthermore, in our model we restricted our analysis to structures that actually occurred in the experiment to keep the model as simple as possible, while still exhibiting the main effect of structure-specific facilitation. In future it might be interesting to model more complex sets of structures.

In this study we employed a very specific notion of stimulus-response learning, namely learning an association between a given sensory representation and a given set of motor responses. However, one might argue that associative learning could also involve more abstract or higher-order representations in the nervous system [38]. Such higher-order associations might even generalize and generate behaviour consistent with structural learning. Such a broad notion of stimulus-response learning is certainly consistent with our results, but crucially would involve a hierarchy of abstraction levels. Such hierarchical organization is a recurring theme in neuroscience. There have even been attempts to identify hierarchical control structures in the brain [39]. In a Bayesian framework hierarchical learning is naturally implemented and captures human learning on multiple scales. Hierarchical Bayesian inference might therefore provide a synthesis between classic ‘telephone switchboard’ accounts of learning and more “insightful” learning based on abstraction and structure learning [14], [15].

## Methods

### Ethics Statement

Twenty naive subjects participated in this study and gave written informed consent after approval of the experimental procedures by the Ethics Committee of the Albert-Ludwig University Freiburg. The subjects were students recruited from the university environment.

### Experimental Procedure

Subjects sat at a computer screen that displayed nine equally sized squares arranged on a 3×3 grid. The stimulus consisted of one of the squares lighting up. Subjects then had to respond by pressing one of nine buttons that were also arranged in a 3×3 grid to encourage the idea of a “geometric” or “spatial” mapping (Figure 1A). If they pressed the correct button they were informed by a high-pitch beep, otherwise there was a low-pitch tone. Then another randomly selected stimulus lit up. There were six possible mappings subjects had to learn: Identity, Right Shift, Left Shift, Vertical Mirror, Horizontal Mirror, and Random (Figure 1B). The shift mappings were circular such that, for example, the right-most button in the right shift would be mapped to the left-most button in the same row. There were two groups of subjects (ten in each group) that learned the mappings in a different order. All subjects started with the identity mapping. Then the first group learned the above mappings in the order: Right Shift, Left Shift, Horizontal Mirror, Vertical Mirror, and Random. The second group had the order of some of the mappings reversed: Left Shift, Right Shift, Vertical Mirror, Horizontal Mirror, and Random. Each mapping was deterministic and bijective, i.e. there was always one response that was uniquely associated with one stimulus. Learning of a mapping was considered successful once the subject had managed to give the right response for each of the 9 stimuli without making any intervening mistakes. Subjects were indicated that the mapping changed thereafter. We counted the number of trials for successful learning of a mapping as an indicator of performance. Subjects were instructed that each of the nine stimulus squares corresponded to exactly one of the nine buttons and that they should find the correct button as quickly as possible. Subjects were not informed about possible structures of the mappings.

### Model 1: Feed-Forward Neural Network

Both the input (x) and output (y) were represented as 9-dimensional binary vectors. The output was given by a linear combination of the inputs, such that 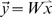. The weights were updated using back-propagation, i.e. 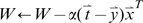, where 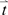 represents the target vector (the correct response). The learning rate was set to 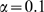. The network was initialized by training the identity mapping. Then the random mapping and the right-shift mapping were learned. We initialized the network with the right-shift mapping when learning the left-shift mapping. Performance was assessed as the number of trials needed for a performance below the error threshold 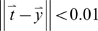.

### Model 2: Reinforcement Learning Model

For each stimulus 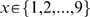 and action 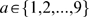 we defined an action value-function 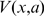. Actions were sampled from this function according to the softmax-rule: 
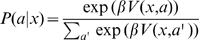
. The parameter 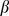 corresponds to the temperature in physical models and regulates exploration. We set 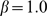. If the sampled action corresponded to the correct response then a reward of 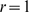 was delivered, otherwise 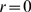. The action value-function was updated using the delta-rule (or Rescorla-Wagner rule), i.e. 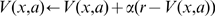. The learning rate was set to 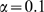. We initialized the action-value function with the identity mapping and then learned both the random mapping and the right-shift. We then initialized the value function with the right-shift before learning the left-shift.

### Model 3: Non-Hierarchical Bayesian Model

The hypothesis set was given by all possible mappings, which could be represented by 9! permutations of the numbers 1 to 9 – the identity mapping, for example, would be 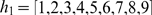, the right-shift mapping 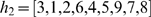, the left-shift mapping 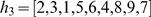, the horizontal mirror mapping 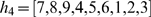, and the vertical mirror mapping 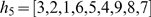 (Fig. 1). The likelihood models were binary such that they assigned the value 1 to all mappings that were compatible with an observation, and zero otherwise:
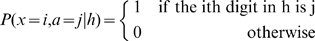
When learning the random mapping and the right-shift mapping, the prior probability was set as follows: 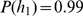, 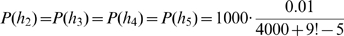 and 
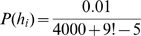
 for 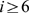. Thus, structured mappings had a thousand times more prior probability than random mappings. When learning the left-shift mapping, the prior probability was assigned mostly to the right-shift mapping such that 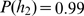 and 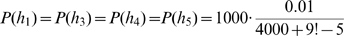 and 
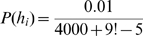
 for 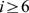 as previously. This was to assess whether learning a right-shift mapping might facilitate learning a left-shift mapping. Actions were chosen stochastically by sampling a hypothesis from the posterior distribution 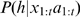 and executing the action suggested by the sampled hypothesis-mapping. This allowed us to model noisy decision making. If the sampled hypothesis corresponded to the true hypothesis learning could proceed much faster because finding the correct answer to a stimulus allows ruling out all other 8 possible answers to the particular stimulus, whereas sampling the incorrect hypothesis only allows eliminating 1 possible answer to that particular stimulus. The prior probabilities were set manually to ensure that all hypotheses had non-zero probability mass at the start of learning.

### Model 4: Hierarchical Bayesian Model

As in the non-hierarchical model, the hypothesis set was given by all possible mappings *h*. Additionally, we introduced four structures that comprised the various hypotheses. The first structure 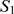 was the ‘identity structure’ with only one member, i.e. the identity mapping 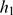. The second structure 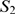 was the ‘shift structure’ that contained both the right-shift and the left-shift mapping (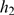 and 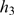). The third structure 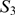 was the ‘mirror structure’ that consisted of horizontal and vertical mirror mapping (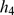 and 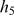). Finally, the fourth structure 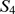 contained all other mappings and is referred to as the ‘random structure’. The likelihood model was the same as in the above model, this time written as 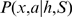. Additionally, we defined the prior probabilities 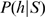 as 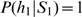, 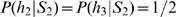, 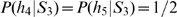 and 
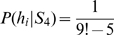
 for 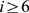. The posterior over hypotheses can then be computed as
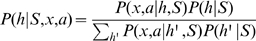
Importantly, in this hierarchical model we can also compute a posterior over the structures:
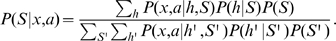
Thus, learning, for example, the right-shift (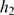) will not only lead to a higher posterior probability of the right shift hypothesis, but also of the shift structure, and therefore can facilitate learning of the left-shift. When learning the random mapping and the right-shift mapping, the prior probability over structures was set as follows: 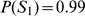, 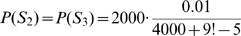 and 
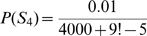
. Thus, structured mappings had a thousand times more prior probability than random mappings. When learning the left-shift mapping, the prior probability of the shift structure was elevated. We set 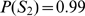, 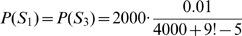 and 
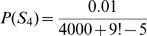
 as previously. Actions were again sampled from the posterior 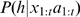, that can be computed as 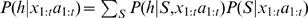.
